# Diagnosis of gluten-related enteropathy in a newborn: how and when? 

**Published:** 2019

**Authors:** Roberto Assandri, Alessandro Montanelli

**Affiliations:** 1 *Departement of Clinical Pathology, Clinical Chemistry Laboratory ASST Ospedale Maggiore di Crema, Italy*; 2 *Department of Health Prevention Clinical Chemistry Laboratory, ATS Bergamo, Italy *

**Keywords:** Diagnostic tests, Transglutaminase antibodies, Deamidated gliadin peptides

## Abstract

**Aim:**

To analyze the development of gliadin-specific immune responses in children with a genetic risk for CD and to determine whether these could be detected before the clinical onset of the disease by using immunological tests.

**Background:**

Clinical manifestations of celiac disease (CD) in the first year of life is uncommon, which is due to the suboptimal sensitivity of tissue transglutaminase IgA antibodies (tTG-IgA) at this age and other possible causes of malabsorption in infants. The development of Deamidate gliadin peptide-specific antibodies (in particular DGP-IgG) in young children was poorly considered in the CD diagnosis.

**Methods:**

We conducted a retrospective cross-sectional study on children between one month and forty-eight months of life, which performed in our health center from 2016 to 2018. Three hundred and fifty children were selected according to strict inclusion criteria: positive for HLA-DQA1 and DQB1 alleles, positive anti tTG-IgA/IgG and/or positive DGP-IgG/IgA. Eighty-two children were selected and divided into two different groups of patients: Group one (forty newborns under twenty-four months of life) and Group two (children from twenty-five months to 48 months of life).

**Results:**

Anti-DGP-IgG antibodies precede anti tTG-IgA seroconversion in children under two years in 80% of cases. Anti-DGP-IgG positive patients had milder symptomatic forms of CD than anti tTG-IgA positive children, characterized by gastrointestinal symptoms in the presence of normal growth, normal serum iron, and low MCH level. At tTG-IgA seroconversion, children present gastrointestinal clinical forms associated with impaired growth. The combined use of tTG-IgA and DGP-IgG antibodies upgrade the diagnostic sensitivity from 50% to 92%.

**Conclusion:**

Anti-DGP-IgG antibodies precede tTG-IgA seroconversion in newborns and identified two distinct clinical phenotypes. At this point, if you wanted to test your newborn patients for CD serology, how would you proceed?

## Introduction

 The Oslo definition criteria considered the celiac disease (CD) a multiple, systemic immune-mediated disorder, triggered by the ingestion of wheat gluten and related proteins in genetically predisposed individuals (1). It is well known that CD is strictly associated with specific human leukocyte antigen (HLA) class II genes, well noted as HLA-DQ2 and HLA-DQ8. These are located on chromosome 6p21, which confers susceptibility to gluten-specific T cells in the gut by the presentation of specific immunogenic gluten peptides (2). HLA-DQA1 and HLADQB1 loci are present in about 90-95% of CD patients with DQ2.5 heterodimers and DQ8 molecules (3).

The gliadin-reactive CD4+ T cells enhance an adaptive immune response that leads to an intraepithelial and lamina propria infiltration of inflammatory cells, crypt hyperplasia and villous atrophy (4). Innate immunity also contributes to mucosal damage (4-7). IL-15 cytokine is rapidly induced after gliadin exposure only in celiac patients (7,8). In the end, the expression of non-classic MHC class I molecules in response to gluten exposure activates CD8+ cytotoxic T cells, which can target and destroy epithelial cells (4).

Although Literature considers intestinal biopsy the diagnostic “gold standard”, the relevance of serology has increased over the years. 

Immunoglobulin A (IgA) antibodies against tissue transglutaminase (tTG-IgA) and endomysium (EmA) are now considered the best markers for CD. 

Deamidated gliadin peptide antibodies (DGP), subclasses IgA, and IgG (DGP-IgA, DGP-IgG) has replaced the traditional antibodies against gliadin (8).

Today DGP–IgG is known to have diagnostic accuracy comparable to tTG-IgA and EmA in patients with CD (8). 

However, little is known about their sensitivity, specificity, and predictive value in infants and children with suspected gluten enteropathy. 

European Society for Pediatric Gastroenterology guideline recommended the use of IgA class antibody tests in IgA-competent subjects and also defined subjects with low serum IgA levels as total serum IgA<20 mg/dL. For these patients, the conclusions should be drawn looking at the results of the IgG class CD-specific antibody tests (9).

However, the literature showed conflicting and weak data. The use of DGP-IgG in infancy and children with suspected gluten enteropathy ranged from a significant predictive value (10) to poor predictive value. It was less sensitive in younger children and less effective in patients with normal IgA level (11).

We aimed to analyze the development of gliadin-specific immune responses in children with a genetic risk for CD and to determine whether these could be detected before the clinical onset of the disease by using immunological tests. 

## Methods

**Study design**

**Figure 1:**
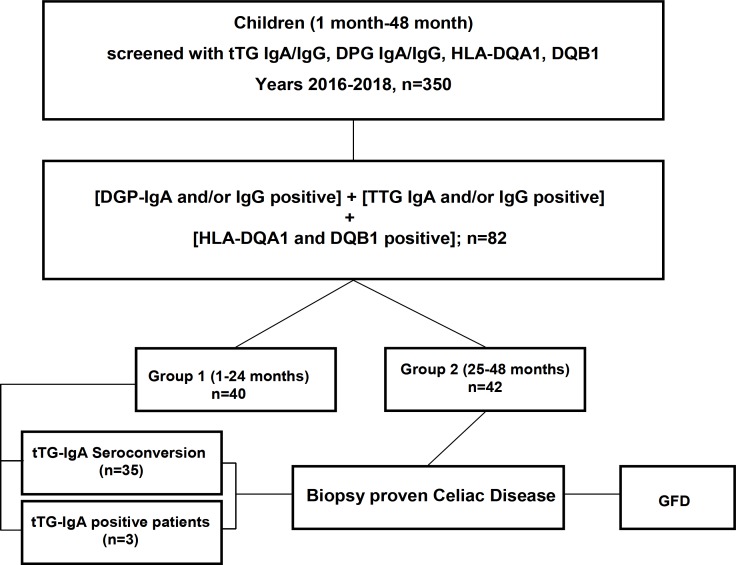
Study design. Retrospective cross-sectional study of children referred to our center during the time spanning 2016–2018 (three years), ranged from one month to 48 month

As illustrated in Figure 1, we performed a retrospective cross-sectional study on children referred to our center from 2016 to2018 (three years), aged between one month and 48 months. A total of 350 children were selected using the following restrictive parameters:

1. DGP-IgA and/or IgG positive

2. TTG IgA and/or IgG positive

3. HLA-DQA1 and DQB1 positive

Selected patients were divided into two groups: 

Group 1: 1 month- 24months

Group 2: 25 month- 48 months 

Patients were excluded in three cases: 1- they had a previous diagnosis of CD; 2- they had a positive TTG-IgA at any time; 3- they were on a gluten-free diet at the time of serological tests. 

**Antibodies assay**

A commercial FEIA-based kit was used to measure tTG-IgA, tTG-IgG, DGP-IgA and IgG (Elia Celikey, Phadia, Freiburg, Germany) with a decisional cut-off value of 7 U/mL for all methods.

**Allergological Workup**

 Serum samples were analyzed for specific IgE antibody titers against wheat and gluten using a commercially available system (Immuno CAP, Phadia 250; Phadia, Uppsala, Sweden). The cut-off values were set for values > 0.10 U/L

**HLA molecular typing**

Polymerase chain reaction (PCR) amplification of extracted DNA followed by hybridization with sequence-specific oligonucleotide probes. Results reported: positive or negative for HLA-DQ2 and -DQ8; specific *DQA1* and *DQB1* variants detected 

**Statistical Analysis**

Patient features were presented as the mean ± SD for continuous variables and as frequencies and percentages for categorical variables.

The associations between the Ab level, laboratory parameters, biopsy, and clinical characteristics were examined in the CD patients using Rank Biserial Correlation analysis. Spearman’s rank correlation coefficients were calculated to assess univariate associations between serum Ab levels and continuous variables. 

We used a significance level of p<0.05 (two-sided) for all the analyses. We calculated the sensitivity, specificity, positive predictive value (PPV) of tTGA-IgA, and DGP-IgG. We reported 95% confidence intervals for each calculated PPV. 

We compared proportions and mean serology values using the Chi-square test and unpaired student t-test, respectively. All reported p values were two-sided. 

## Results

**Two patients groups are two distinct clinical Phenotypes**

Table 1 indicates the baseline characteristics of CD patients. Clinical manifestations (intestinal and extra-intestinal symptoms) were typical for CD. Iron deficiency anemia and diarrhea were the most common clinical symptoms, affecting 60.2 % and 55.3 % of the cohort.

The intestinal manifestation was the most relevant clinical symptoms in children under two years (90%), characterized by a pre-anemic condition with MCH level 24.9±2.94 pg (normal range 26-35 pg)

Conversely, extra-intestinal manifestations appeared only in Group 2 (Table 1). Anaemia also appeared in 75% of patients, linked to impaired growth and diarrhea as the most common clinical features. 

Stool test was applied for all patients: Stool cultures were performed for enteric pathogens including Salmonella, Shigella, *Klebsiella oxytoca,* and *Yersinia*. Rotavirus and Adenovirus antigens were searched by immunochromatographic test.

We noted that 90% of patients with pathological DGP -IgG levels were positive for Rotavirus antigen before serological tests. 

Serum samples were analyzed for specific IgE antibody titers against wheat and gluten using a commercially available system. All patients were negative.

**DGP-IgG serum levels were different in two patients groups**

Table 2 summarized serological and biochemical evidence in two groups.

**Figure 2:**
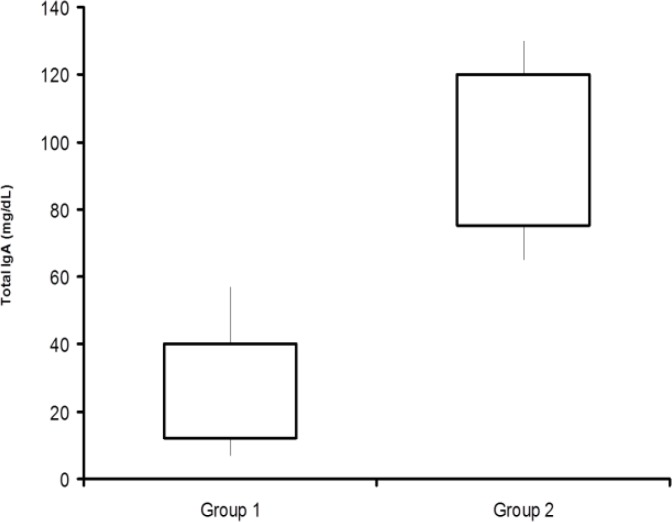
Total IgA levels in two groups

We noted that only five patients in group 1 had a total IgA level under 20 mg/dL. Mean Total IgA level was significantly higher in Group 2 than Group 1 (mean±SD: 106.54±42.57mg/dl Group 2 vs. 38.84±24.79 Group 1, p<0.05, Figure 2). 

**Figure 3:**
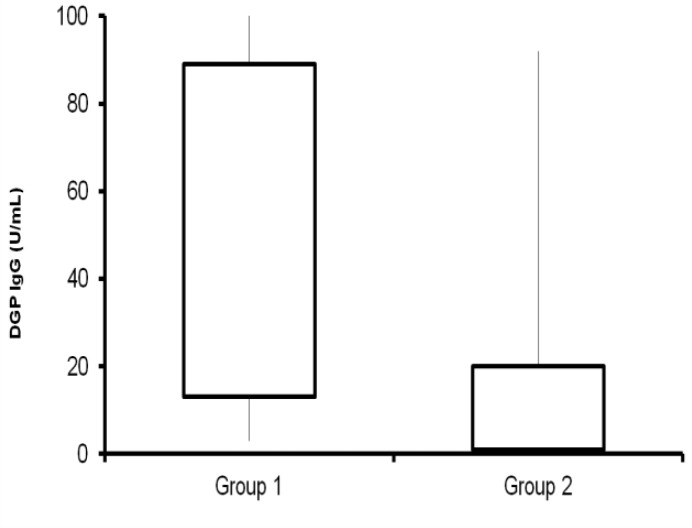
DGP-IgG levels in two groups

The same statistically significant differences were noted between Group 1and 2 for tTG-IgA serum levels (0.76±0.56 UI/mL; 103±28 UI/mL respectively p<0.05) and DGP-IgG serum levels (38.50±10.5 UI/mL 14.4±3.3 UI/mL, respectively. (Figure 3). DGP -IgG was found in 37 (92.5 %) of the 40 (Group 1) children with malabsorption without association with one or more of the other antibodies. 

Our patients were characterized by a mean IgA level of 38.84 mg/dL, and only five patients with IgA level lower than 20 mg/dL. 

**Table 1:** Patients clinical features

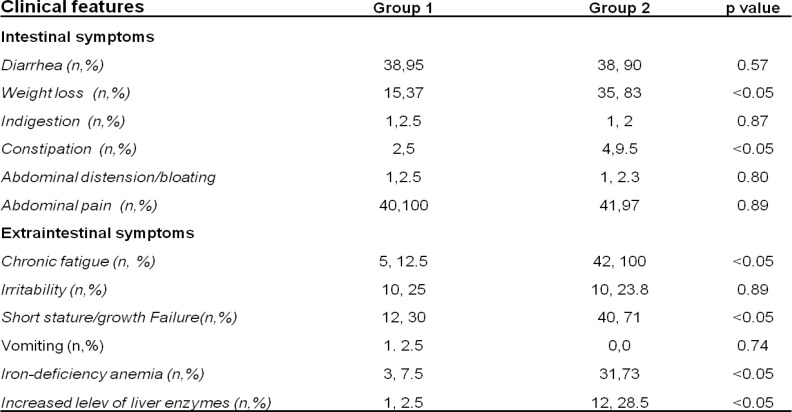

On the thirty-seven patients, the remaining three cases were positive for tTGA, DGP IgA, and EMA. In Group 2 only two patients were found with isolated positivity for DGP-IgG. 

All patients were HLA positive, as showed in the patients and Methods paragraph. More than half of the patients (94%) were considered positive for HLA DQA1, DQB1 (DQA1*05 and DQB1*02). Five patients (three in group 1 and two in group 2) were characterized by DQB1*02:02 DQA1*02:01 alleles.

**Table 2:** Baseline features of CD patients

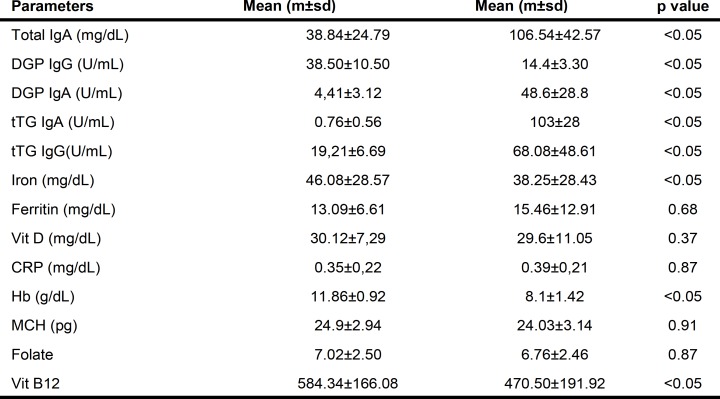

**DGP-IgG precede tTG-IgA seroconversion in children**

tTG-IgA and DGP-IgG were determined at all sampling points starting from two months after first DGP-IgG evidence., theirty five children turned seropositive for tTG-IgA (87.5 %) during follow-up. The mean age of seroconversion was 21 months (range 19–38 months), and the time of seroconversion after the first evidence was eight months. 

Three tTG-IgA positive patients and thirty-seven patients who were positive for DGP-IgG (only after tTG-IgA seroconversion) underwent a duodenal biopsy in group 1. Thirty-three of these children were diagnosed with CD patients (symptomatic-biopsy proven/symptomatic-potential CD patients 94%). Five of them showed villous atrophy (specifically two patients had severe intestinal damage, grade 3b/3c) and Twenty patients showed a Marsh grade 1-2 lesion. 

Forty-two patients in Group 2 underwent a duodenal biopsy. Twenty-two of them showed villous atrophy (specifically two patients had severe intestinal damage, grade 3b/3c). Two patients who were positive for DGP-IgG showed a Marsh grade 1-2 lesion.

**The use of DGP-IgG increased the sensibility and predictive value in CD diagnosis**

The next step consists in identifying the assay's performance characteristics. Estimated values of diagnostic sensitivity (SN), diagnostic specificity (SP), and Positive Predictive Value (PPV) are the primary parameters obtained during the evaluation of tests. SN measures the proportion of actual positive values that are correctly identified. We evaluated SN, SP, and PV of tTG-IgA in two Groups. 

In group 1 tTG-IgA failed in identifying new CD patients (symptomatic-biopsy proven/symptomatic-potential CD patients), with SN as 50% and PPV as 75%. SN and PPV increased after the combined use of tTG-IgA/DGP-IgG in patients under 24 months of life (from 50% to 92% and from 75% to 97%, respectively). 

In group 2, the only use of tTG-IgA as biomarker showed an SN as 92%. The tTG-IgA/DGP-IgG mixed-use increased the SN tp95%.

**Nutritional status: Iron, Vit D, Folate and VitB12 deficiency were the effects of gluten contained diet**

The inflammation in CD leads to malabsorption of essential nutrients. Iron, 25-hydroxyvitamin D (VitD), cobalamin (VitB12), and Folate were evaluated in all patients. Children in group 2 had nutritional deficiencies (as reported in Table 2). The same condition does not appear in group 1 (< 2a, see Table 2). 

We demonstrated that a deterring iron level and VitB12 occurred with a tTG-IgA seroconversion. Iron malabsorption was evident after 12 months on a gluten-containing diet.

Patients under two years of life were characterized by a low VitD serum level (mean at 30.12 mg/dL) like CD patients in Group 2 (mean at 29.6 mg/dL).

## Discussion

Literature showed conflicting and inadequate data about the use of DGP-IgG. 

The diagnostic rule of DGP-IgG in infancy and children with suspected gluten enteropathy ranged from important predictive values (10) to poor predictive values (11).

In this study, the lack of tTG-IgA with the only presence of DGP-IgG are the typical serological manifestations in children under two years of life Intestinal manifestations were the most relevant clinical symptoms in children under two years (90%), characterized by a “pre-anaemic” condition with low MCH level (24.9±2.94; normal range 26-35 pg). 

Anaemia also appeared in 75% of patients, linked to impaired growth and diarrhea as the most presenting clinical features in patients >2 years (Table 1). DGP-IgG appeared as a real serological marker in children under 24 months. 

The combined use of DGP-IgG and tTg-IgA also increased the diagnostic sensibility and specificity of tTGA (50% to 92%).

The impact of these evidence during the steps of CD diagnosis is significant.

Indeed, the Paediatric guideline, written in 2012 recommended DGP-IgG test only in selected groups of patients with a low IgA total level (<20mg/dL) (9). Our patients were characterized by a mean IgA level of 38.84 mg/dL and only five patients by an IgA level lower than 20 mg/dL. 

In a large Finnish DIPP follow-up study, the earliest seropositivity to tTG-IgA has been reported at the age of 12 months (12). In this study, it seems that tTG-IgA seropositivity in children younger than two years is extremely rare. Several studies have demonstrated that DGP antibodies perform high sensitivity and specificity in the diagnosis of CD, both in adults and children (13-15).

Gould and colleges conversely affirm DGP-IgG should not be completed as part of the initial screening for celiac disease in IgA sufficient individuals (10). The author conducted a multicenter retrospective review of children, from birth to age 18. For this reason, these data are not comparable with ours, because of the use of an “extra-large” patients cohort, poorly in newborn and children under two years of life (10).

In our study, more than half of the children had high DGP-IgG before tTG-IgA seroconversion. 

The mean age at seroconversion was 21 months (range 19–38 months) similar evidence was reported in 2016 by Lammi and colleagues (16). They correlated their results to previous retrospective studies, which demonstrated that DGP-IgG seropositivity precedes tTG-IgA-seropositivity (17,18). 

Estimates of diagnostic sensitivity (SN), diagnostic specificity (SP), and Positive Predictive Value (PPV) are the primary parameters obtained during the evaluation of tests. SN (also called as the right positive rate) measures the proportion of actual positives that are correctly identified. Therefore, DGP-IgG antibodies have a good sensitivity in CD evaluation. 

About 90-95% of CD patients carried DQ2.5 heterodimers, which were encoded by DQA1*05 and DQB1*02 alleles both in cis and in Trans configuration and DQ8 molecules, encoded by DQB1*03:02 generally in combination with DQA1*03 variant (19).

HLA molecular typing for CD is a genetic test with a negative predictive value (NPV). Nevertheless, it is a vital tool able to discriminate individuals genetically susceptible to CD (20, 21).

Volta and colleagues discussed the role of DGP-IgG in CD diagnosis and emphasized the relationship between DGP-IgG and biopsy status in newborn CD patients. Authors showed that high DGP titers are closely related to severe intestinal damage. In line with the relevance of CD-antibody titers, stated by ESPGHAN for tTG-IgA, a high DGP titer in addition to HLA-DQ2/DQ8 positivity may represent a diagnostic alternative to biopsy in infancy (8).

In our study, we demonstrated that DGP-IgA failed to detect CD pathological conditions in Group 1 patients.

In conclusion, IgA lineage is not primarily involved in the first step of CD condition.

This idea is strongly linked to the expression of gliadin IgG antibodies in NCGS. Innate immunity is also considered the possible first disease pathway, involved in CD development (5-7).

Based on our experiences and our findings, we propose the DGP -IgG detection in all patients less than two years of life with suspected gluten enteropathy, despite IgA levels.

In our opinion, it is necessary to start GFD when clinical symptoms and DGP-IgG appeared in HLA-DQA1/B1 genetic subset.

The inflammation in CD leads to the malabsorption of essential nutrients. This pathological situation should improve by the adherence to aGFD.

In patients with CD, nutritional markers should be evaluated at diagnosis, and abnormal findings should be re-evaluated after one year of GFD. Wessels et al. showed that 28% of children with CD at diagnosis have a nutritional deficiency, such as iron (28%), folate (14%), VitB12 (1%), or VitD (27%) deficiency (21). As showed, our children in group 2 (> 2 years old) had nutritional deficiencies as reported in Literature. The same condition does not appear in group 1 (< 2 years old patients). tTG-IgA seroconversion occurred a deteriorating iron VitB12 and VitD status. 

More than 25% of the world’s population is affected by anemia, and more than 50% suffer from iron deficiency anemia (22). Children under seven years of life are part of the population who is most vulnerable to iron deficiency. Iron is an essential element in brain metabolism. Iron deficiency can cause changes in neurotransmitter homeostasis, decrease myelin production, impair synaptogenesis, and decline the function of the basal ganglia (22). VitD deficiency is associated with a wide range of chronic diseases and conditions, including obesity, and with increasing severity of metabolic deregulation such as insulin resistance, hyperlipidemia, and liver diseases (23).

These results lead us to ask two questions: Why does tTGA Abs fail to intercept newborn patients? What is the molecular pathway involved in DGP-IgG expression?

The immature IgA subset and their related mediator cells are the first hypotheses. Gliadins are the major pathogenic elements s of wheat; their toxicity and immunogenicity depend on their amino acid sequence. They initiate the innate and the adaptive immune response. Nevertheless, it is not known yet how the intestinal epithelium and immune cells recognize them. 

Toll-like receptors (TLRs) are the best-studied groups of pattern recognition receptors, which play a critical role in the initiation of innate immunity through the recognition of pathogen- and damage-associated molecular patterns. Gliadin can activate TLRs signaling pathway in vitro. The existing evidence is suggestive of the direct contribution of gliadin and/or other wheat components to enabling TLRs signaling (19). Gliadin could be a direct ligand for one of the TLRs (24). Also, viral infections are now considered as possible trigger factors for CD condition. 

Our patients were characterized by a Rotavirus infection evidence, which was indicated before the first evidence of CD serology markers. TLR7 is an endosomal receptor that specifically recognizes the viral mRNA and is regulated by endosomal trafficking (25). The signaling activated complex induces the phosphorylation of mitogen-activated protein kinase (MAPK), nuclear factor-κB (NF-κB) activation and increasing IFN-α levels (25-28).

Based on the results presented here, viral infections and alimentary proteins, which can mimic and potentiate the innate immune response to viruses, trigger an autoimmune disease (5-7). In addition to CD, other autoimmune diseases result from the interactions of several factors, including genetics and the environment. Type 1 interferon activation is linked to autoimmunity not only in CD but also in several other human diseases, including type 1 diabetes (29). In the end, we can say that our data bring light to two distinct phenotypes of disease:

- Phenotype 1: patients under 24 months of life with DGP-IgG as an only serological marker. These patients were characterized by intestinal manifestations and normal Iron level, without anemia.
- Phenotype 2: patients were characterized by extraintestinal manifestation, impaired growth, and anemia with tTG-IgA as a primary serological marker. Now, if you wanted to test your newborn patient for CD serology, how would you proceed?.

## Conflict of interests

The authors declare that they have no conflict of interest.
